# Supplementary material for: Ternary molecular switching in a single-crystal optical actuator with correlated crystal strain
Source: Nat Commun. 2025 Feb 11;16:1546. doi: 10.1038/s41467-025-56795-w (PMC11814404; doi:10.1038/s41467-025-56795-w)
Supplement: Supplementary file 1 — Supplementary Information [file 41467_2025_56795_MOESM1_ESM.pdf]

**Supplementary Information for:**

# Ternary molecular switching in a single-crystal optical actuator with correlated crystal strain

Jacqueline M. Cole,[1,2,3,4*] David J. Gosztola,[3] Jose de J. Velazquez-Garcia,[1] Jeffrey R. Guest[3]

[1] Cavendish Laboratory, Department of Physics, University of Cambridge, J. J. Thomson Avenue, Cambridge, CB3 0HE, UK.

[2] ISIS Neutron and Muon Source, STFC Rutherford Appleton Laboratory, Harwell Science and Innovation Campus, Didcot, OX11 0QX, UK.

[3] Center for Nanoscale Materials, Argonne National Laboratory, 9700 South Cass Avenue, Lemont, Illinois 60439, United States

[4] Materials Science Division, Argonne National Laboratory, 9700 South Cass Avenue, Lemont, Illinois 60439, United States

* Author for correspondence (J. M. Cole): jmc61@cam.ac.uk

## Table of Contents

Supplementary Note 1:

Single-crystal optical absorption spectra and microscopy images of **1** .................. S-2
(a) Single-crystal optical absorption spectra of **1** illustrating the stability of its photoisomerised states as a function of time and temperature ...................... S-2
(b) Single-crystal optical microscopy images of **1** exposed to 2 h of 505 nm light and then heated progressively to 260 K .................................................. S-4

Supplementary Note 2:

Extended details about the atomic force microscopy (AFM) experiment .............. S-5
(a) Root-mean-square height across the *in-situ* atomic force microscopy images of **1** collected as a function of progressive light exposure or heating .............. S-5
(b) Crystals of **1** glued onto a sapphire disk shown before and after the *in-situ* atomic force microscopy experiment ....................................................................... S-6
(c) Technical checks to validate the efficacy of the AFM data ............................... S-7

Supplementary Note 3:

Hirshfeld surfaces of dark- and light-induced states of **1** at 100 K or 90 K ............. S-8

Supplementary Note 4:

Crystallographic Information Files (CIFs) ................................................................. (see separate files)
(a) CIF for the dark-state crystal structure of **1** acquired at 100 K .......................... (see separate file)
(b) CIF for the light-induced crystal structure of **1** acquired at 90 K ....................... (see separate file)
(c) CIF for the light-induced crystal structure of **1** acquired at 100 K ..................... (see separate file)
(d) CheckCIF report for the dark-state crystal structure of **1** at 100 K .................... (see separate file)
(e) CheckCIF report for the light-induced crystal structure of **1** at 90 K ................. (see separate file)
(f) CheckCIF report for the light-induced crystal structure of **1** at 100 K ............... (see separate file)

**Supplementary Note 1: Single-crystal optical absorption spectroscopy and microscopy images of 1**

**(a) Single-crystal optical absorption spectra of 1 illustrating the stability of its photoisomerised states as a function of time and temperature**

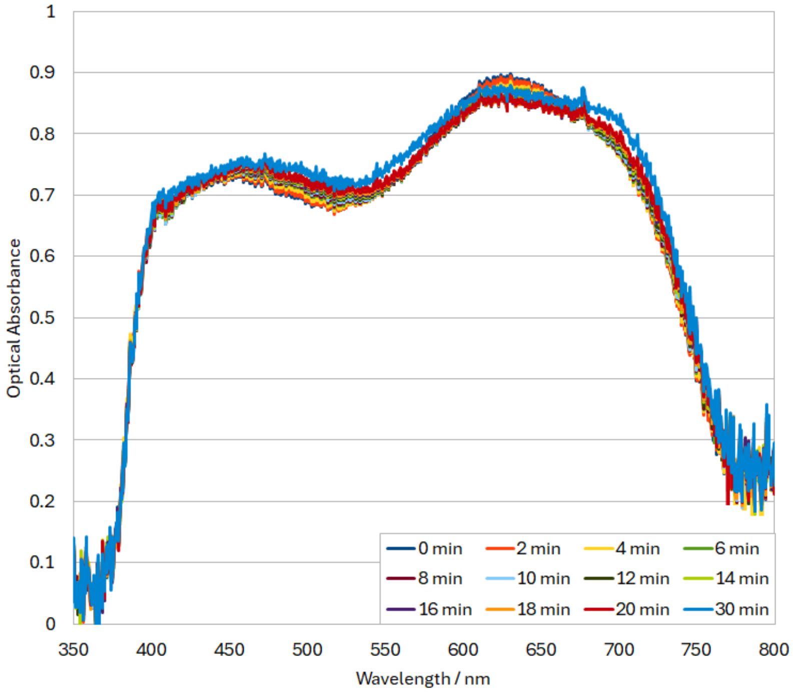


***Figure S1** - **Single-crystal optical-absorption spectra of a crystal of 1 acquired t minutes after the crystal had been stimulated with 505 nm light for 2 h at 90 K**, to monitor its stability at 90 K once the 505 nm light had been extinguished.*

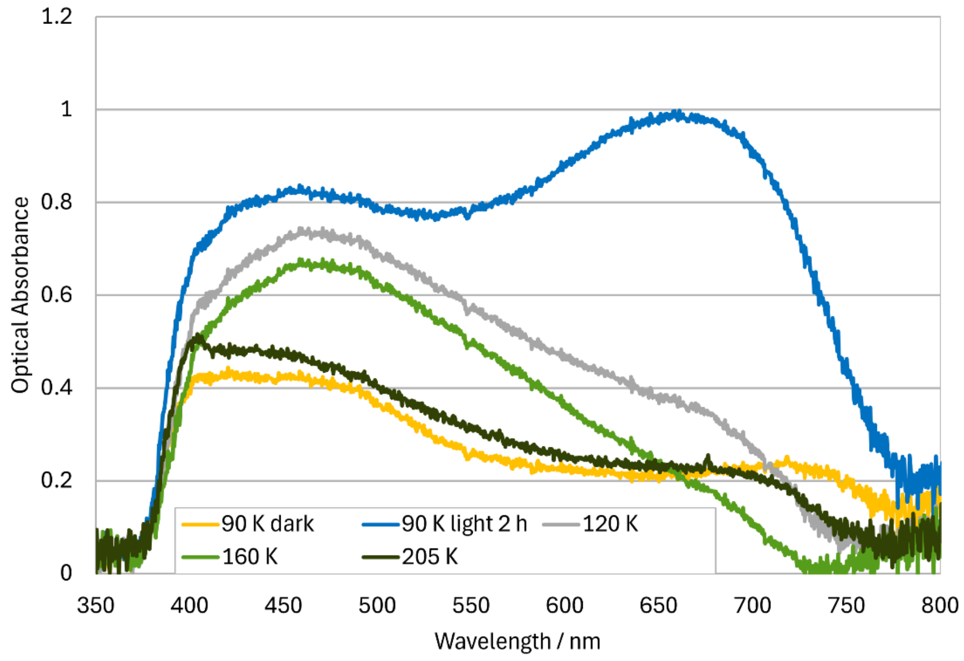


***Figure S2 - Single-crystal optical-absorption spectra of a crystal of 1 acquired under a sequence of light and temperature conditions****: before and after the crystal had been stimulated with 505 nm light for 2 h at 90 K, and then, having extinguished the light, heating the crystal progressively to room temperature at 3 K per minute stopping for 2 minutes at 120 K, 160 K and 205 K for recording spectra.*

**(b) Single-crystal optical microscopy images of 1 exposed to 2 h of 505 nm light and then heated progressively to 260 K.**

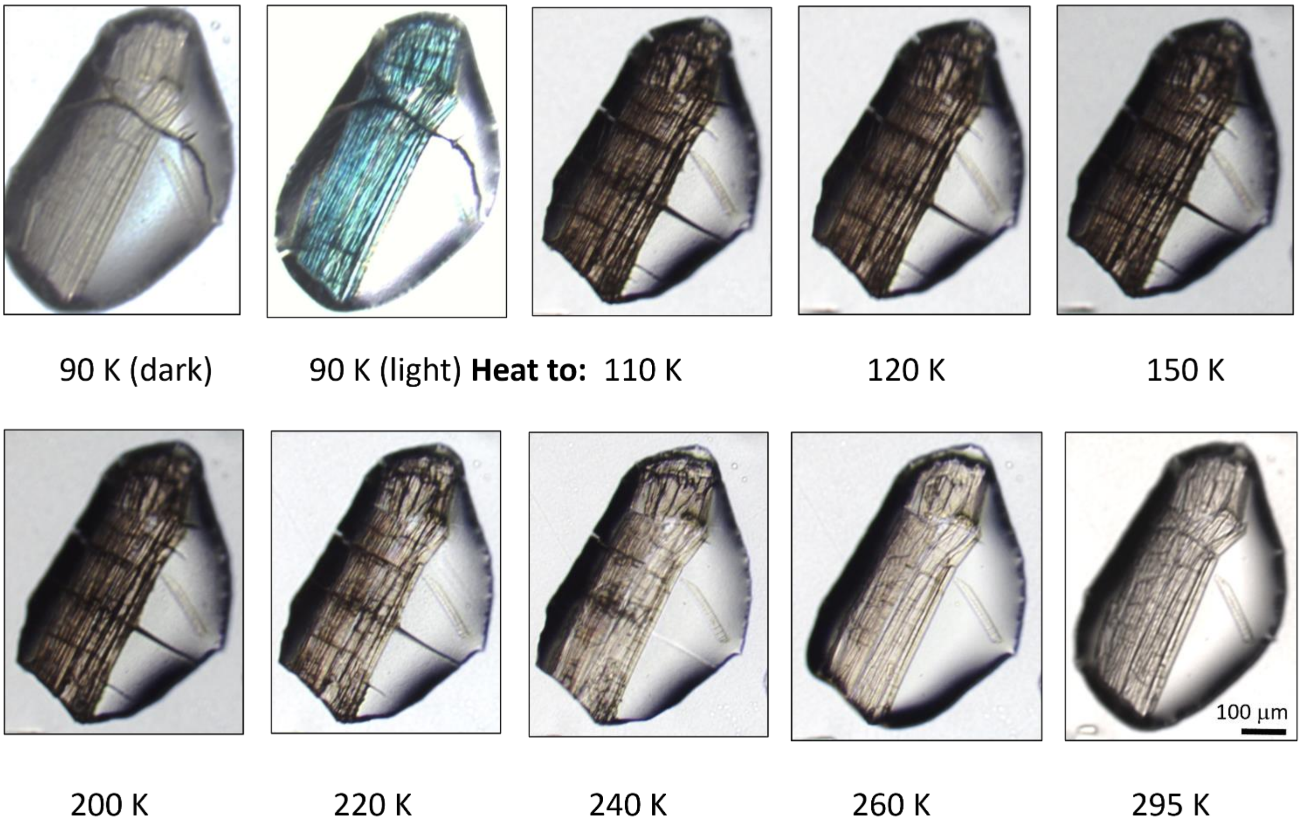


***Figure S3 - Transmission optical microscopy images of a single-crystal of 1 in oil, acquired under a sequence of light and temperature conditions (from top left to bottom right):** first at 90 K (dark) whereupon the crystal is exposed to 2 h of 505 nm light, and is then heated progressively in 8 temperature increments to 110 K, 120 K, 150 K, 200 K, 220 K, 240 K, 260 K and 295 K. The contrast of the image at 295 K is slightly different to the other images because 260 K is the upper limit in temperature when the oil that encloses the crystal is sufficiently viscous to firmly fix the crystal to the backing substrate.*

## Supplementary Note 2: Extended details about the atomic force microscopy (AFM) experiment

### (a) Root-mean-square height across the *in-situ* atomic force microscopy images of 1 collected as a function of progressive light exposure or heating

The root-mean-square (RMS) height across the AFM images was calculated and used as a proxy to quantify the overall extent by which light-induced cracks form in crystals of **1**. Figures S3a and S3b illustrate how this RMS height varies as a function of progressive light exposure below 90 K or progressive warming up to room temperature, respectively.

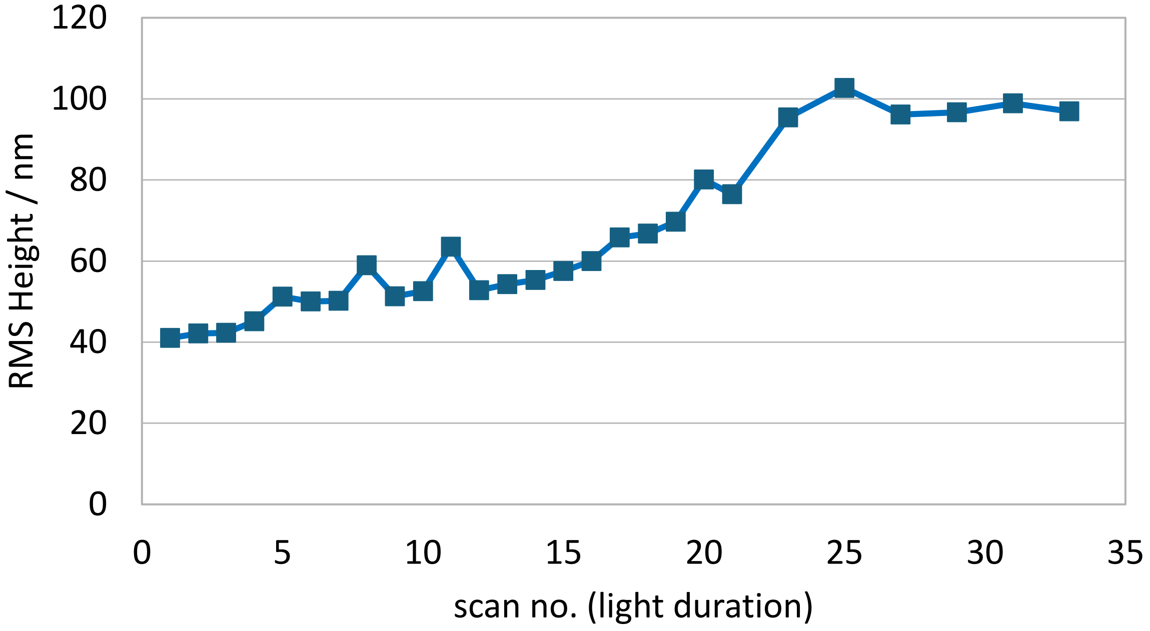


**(a)**

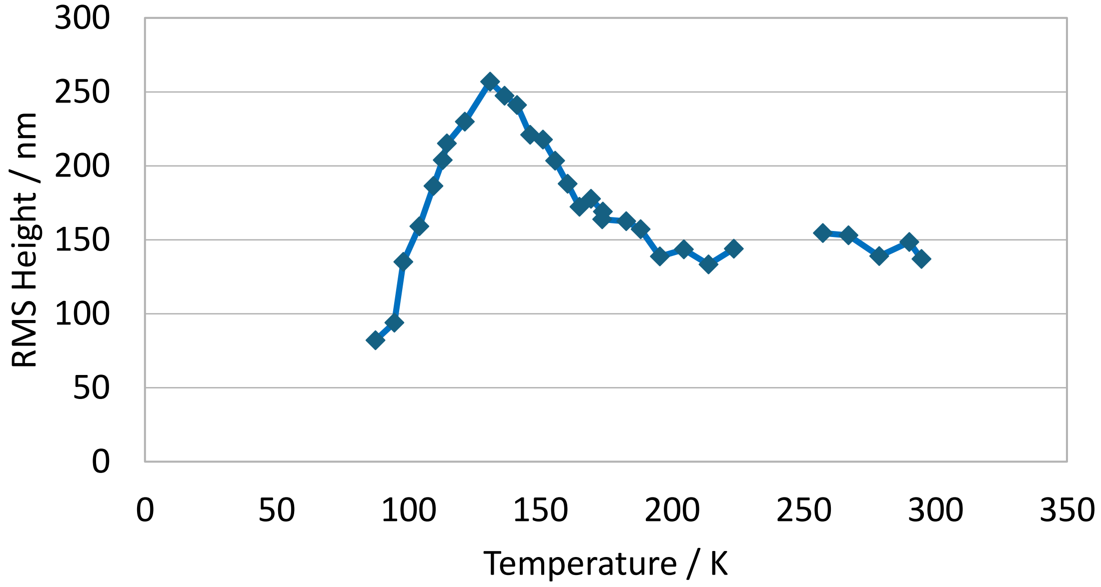


**(b)**

***Figure S4** – **Root-mean square (RMS) height across the atomic force microscopy images that survey the crystal surface of 1 as a function of light and temperature**: (a) progressive exposure to 505 nm light below 90 K; (b) increasing temperature from 87.5 – 295 K.*

**(b) Crystals of 1 glued onto a sapphire disk shown before and after the *in-situ* atomic force microscopy experiment**

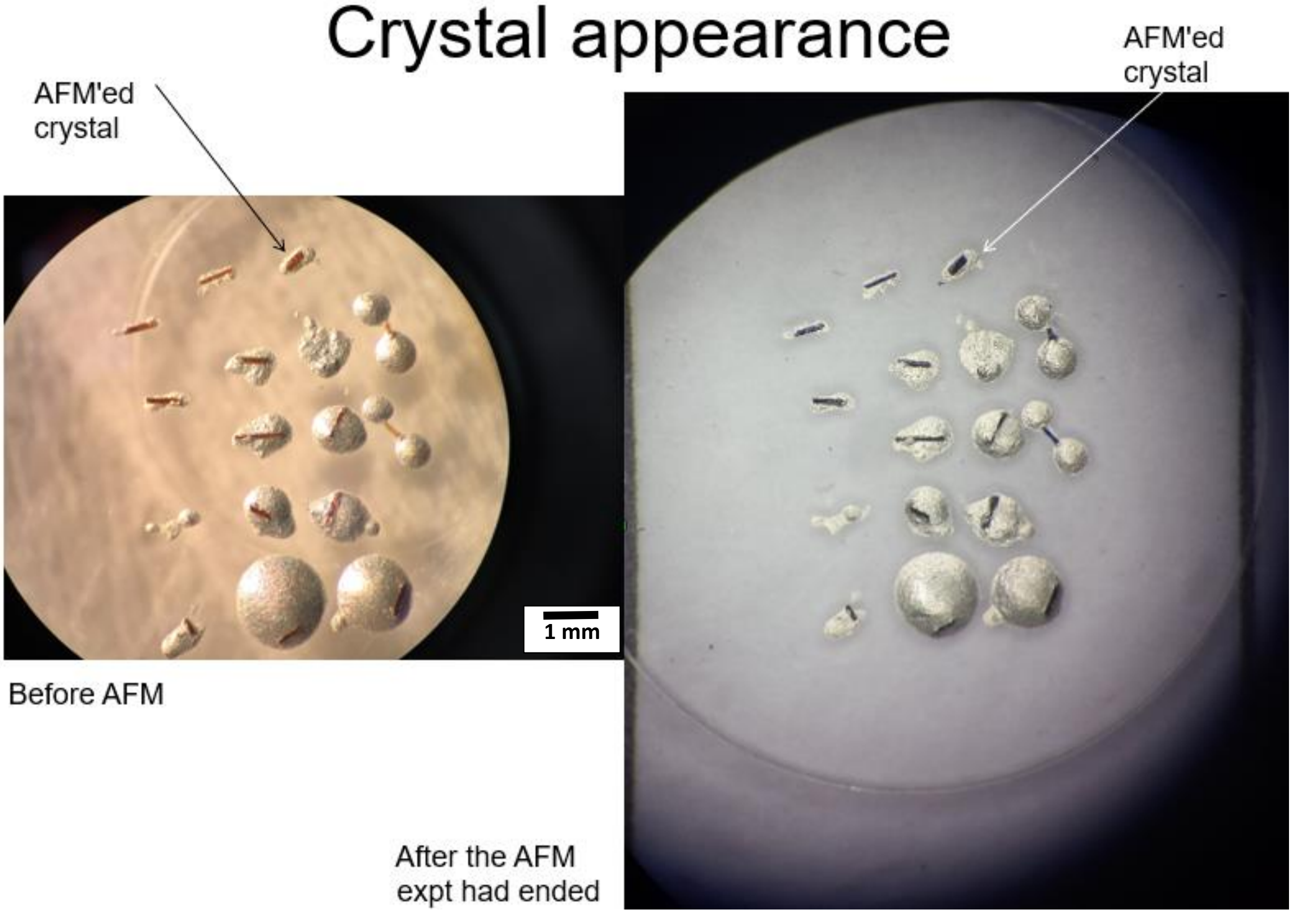


***Figure S5** – **Crystals of 1 glued onto a sapphire disk for the in-situ atomic force microscopy experiment.** The crystals are displayed (left) before and (right) after the AFM experiment. The crystal used for the AFM results presented in this work is highlighted by arrows. All crystals were exposed to cooling and 510 nm light given that the sapphire disk was housed wholly within the AFM vacuum area and the 510 nm laser light was applied at a glancing angle, as required given the restricted geometry of the cryostat windows and physical restrictions of the immediate AFM sample environment. All crystals changed colour to the turquoise-blue hue that is characteristic of the $\eta^1$-OSO photoisomeric crystal structure of **1**.*

## (c) Technical checks to validate the efficacy of the AFM data

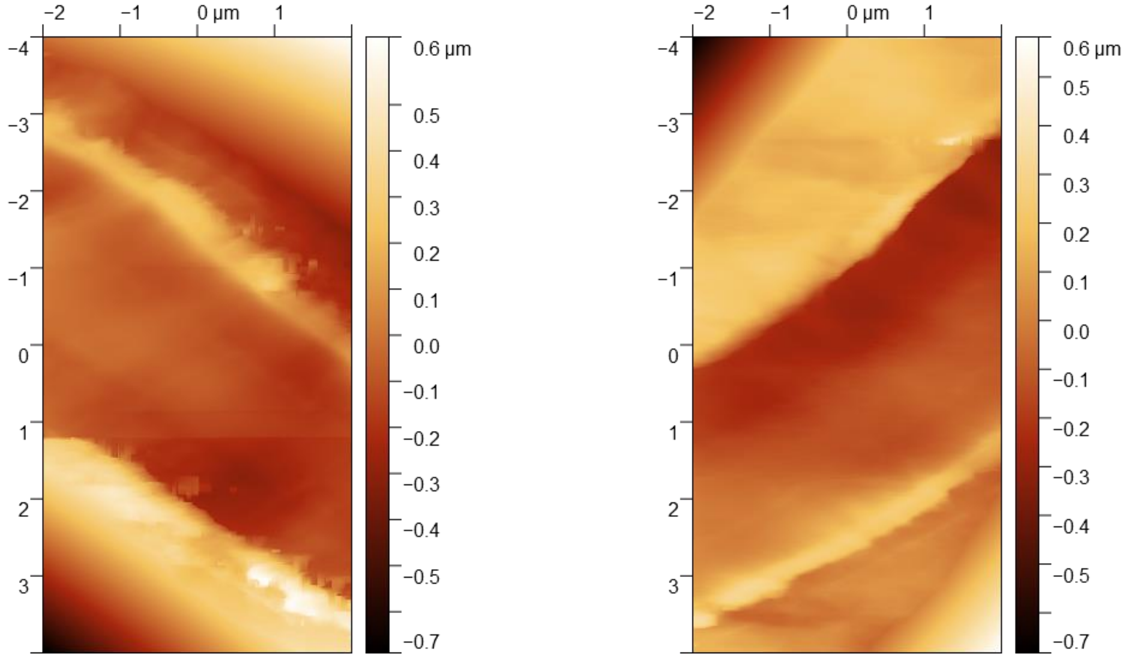


***Figure S6** – **Technical checks for atomic force microscopy**, whereby two AFM images were acquired by directing the AFM tip (left) 45° left and (right) 45° right along the crystal surface of **1**. Symmetric curves with opposing gradients shown in the images confirm that the tip scanned images correctly.*

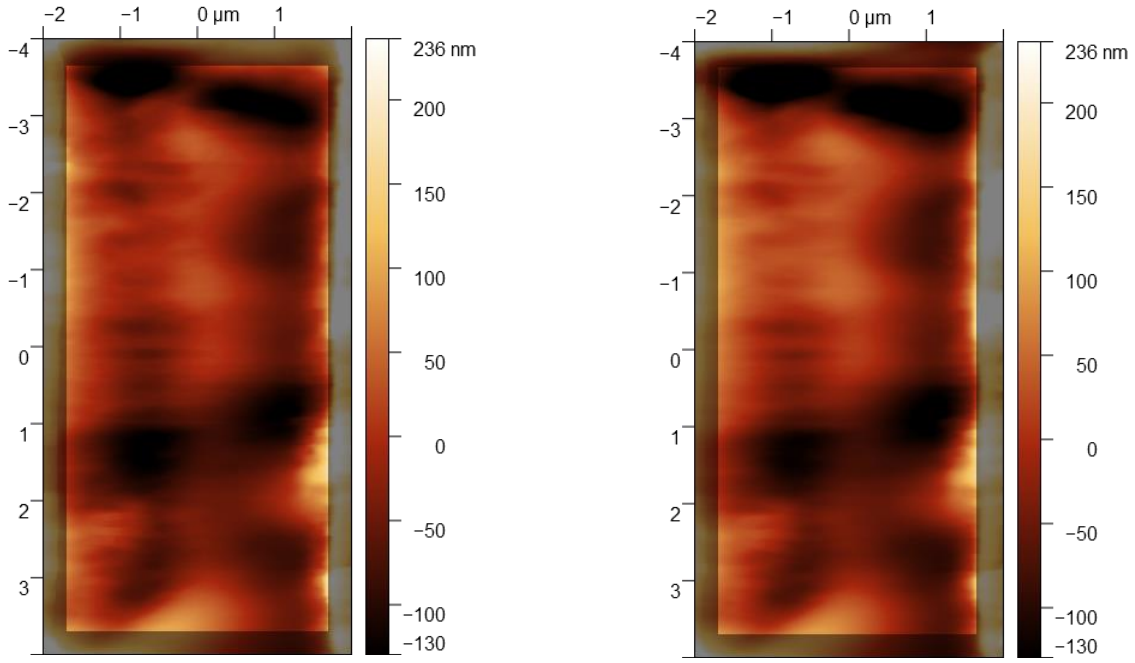


***Figure S7** – **Metastability checks for the photoisomerised state of 1**, whereby two AFM images were acquired following the full 900 min of 510 nm light exposure on the crystal, having extinguished the light and waited to acquire another AFM image for (left) 30 mins; (right) 100 min. The fact that both images reproduce the features of the 900 min light exposed AFM image confirm that the $\eta^1$-OSO photoisomeric crystal structure is metastable once light-stimulated when kept cool below 90 K.*

## Supplementary Note 3: Hirshfeld surfaces of dark and light-induced states of 1 at 100 K or 90 K

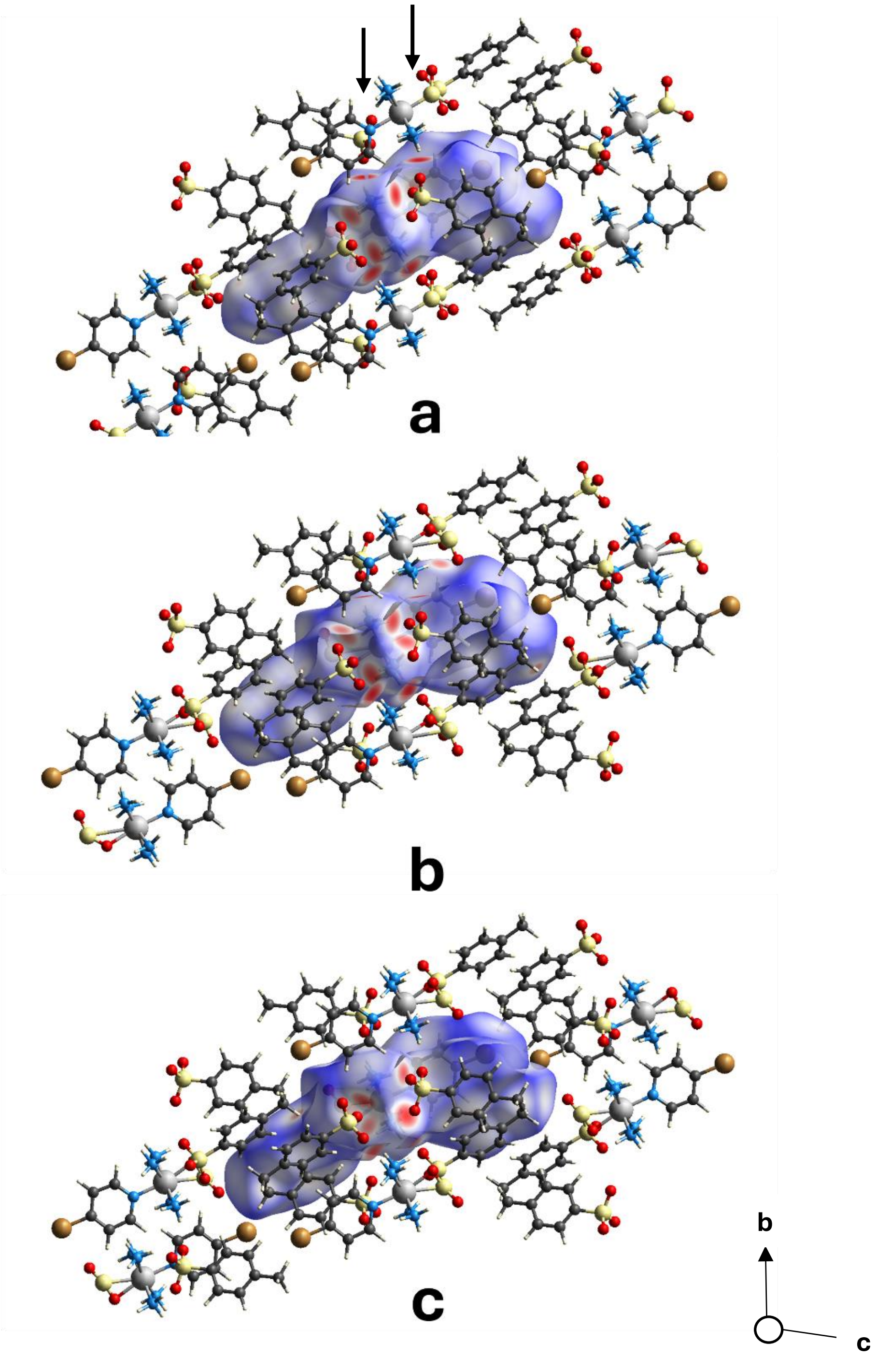


***Figure S8** – **Hirshfeld surfaces of the (a) $\eta^1$-$SO_2$ dark-state, (b) $\eta^2$-(OS)O and (c) $\eta^1$-OSO light-induced crystal structures of 1, looking down the crystallographic axis, a**. The red, white and blue regions show positive, neutral and negative isoenergies. Images were created via CrystalExplorer17 (2017). https://hirshfeldsurface.net. Light exposure depletes two strong steric forces between ammine ligands and counterions that are indicated in red by the two black arrows annotated to the dark-state Hirshfeld surface in (a). The blue volume of the Hirshfeld surface for the dark-state species (691 Å$^3$) contracts to 680 Å$^3$ with light exposure at 90 K while its surface area (554 Å$^2$) increases to 559 Å$^2$; i.e., the Hirshfeld surface is stretched upon light exposure which strains the reaction cavity.*
