# Supplementary material for: Ternary molecular switching in a single-crystal optical actuator with correlated crystal strain
Source: Nat Commun. 2025 Feb 11;16:1546. doi: 10.1038/s41467-025-56795-w (PMC11814404; doi:10.1038/s41467-025-56795-w)
Supplement: Supplementary file 2 — Description of Additional Supplementary Files [file 41467_2025_56795_MOESM2_ESM.pdf]

Description of Additional Supplementary Files

File name: Supplementary Movie 1
Description: A series of single-crystal optical microscopy images showing the thermal stability of a photoisomerised crystal of **1** at 90 K over a 60 min waiting period, having been subjected to 2 h of 505 nm light at 90 K.

File name: Supplementary Movie 2
Description: A series of single-crystal optical microscopy images showing the stability of a photoisomerised crystal of **1** at 105 K over 90 min, having been exposed to 2 h of 505 nm light at 90 K, then heated to 105 K.

File name: Supplementary Movie 3
Description: A series of single-crystal optical microscopy images showing the thermal decay of a photoisomerised crystal of **1** having been exposed to 2 h of 505 nm light at 90 K and progressively warmed to 275 K.

File name: Supplementary Movie 4
Description: Low-temperature *in-situ* atomic force microscopy of a 4 x 8 μm surface area of the single crystal of **1**, illustrating how a crack forms in the crystal while held below 90 K as a result of its progressive 510 nm light exposure.

File name: Supplementary Movie 5
Description: Low-temperature *in-situ* atomic force microscopy of a 4 x 8 μm surface area of the single crystal of **1**, illustrating the depletion of its crystal-cracking effect as a result of progressively warming the crystal from 87.5 to 295 K.
